# CircFRRS1 drives neuroinflammation through the miR-27a-3p/TLR4 pathway after deep hypothermic circulatory arrest

**DOI:** 10.3389/fncel.2026.1750887

**Published:** 2026-02-25

**Authors:** Weidong Yan, Tianlong Wang, Shuai Zhang, Mingru Zhang, Jing Wang, Han Zhang, Jieru Zhang, Ziyu Xie, Bingyang Ji, Changwei Wei

**Affiliations:** 1 Department of Anesthesiology, Beijing Chao-Yang Hospital, Capital Medical University, Beijing, China; 2 Department of Cardiopulmonary Bypass, National Center for Cardiovascular Disease and Fuwai Hospital, Chinese Academy of Medical Sciences, Peking Union Medical College, Beijing, China; 3 Songjiang Research Institute, Songjiang Hospital Affiliated to Shanghai Jiao Tong University School of Medicine, Shanghai, China; 4 Department of Anesthesiology, Beijing Tongren Hospital, Capital Medical University, Beijing, China

**Keywords:** animal model, cardiopulmonary bypass, circRNA, deep hypothermic circulatory arrest, neural injury

## Abstract

Neurologic injury remains a critical complication of deep hypothermic circulatory arrest (DHCA) in aortic arch surgery, with neuroinflammation driven by multiple factors in its pathogenesis. While circular RNAs (circRNAs) are known to modulate inflammatory responses, their specific role in DHCA-associated brain injury has not been established. In this study, we demonstrated that circFRRS1 exacerbates hippocampal neuroinflammation via the miR-27a-3p/TLR4 axis through integrated *in vivo* and *in vitro* approaches. In a rat model of DHCA, machine learning-based motion sequencing (MoSeq) identified delirium-like behaviors, accompanied by hippocampal neuronal necrosis and activation of NLRP3 inflammasome. circFRRS1 was significantly upregulated in hippocampal tissue following DHCA and in hypoxic-ischemic PC-12 cells. Silencing circFRRS1 attenuated oxygen-glucose deprivation/reperfusion (OGD/R)-induced cytotoxicity and suppressed the TLR4/NF-κB/NLRP3 signaling pathway. Mechanistically, circFRRS1 acts as a molecular sponge for miR-27a-3p, thereby relieving its repression of TLR4; inhibition of miR-27a-3p abolished the observed neuroprotective effects. This study identifies circFRRS1 as the first reported circRNA to regulate DHCA-induced neuroinflammation, uncovering a novel epigenetic mechanism and suggesting the potential of circRNA-targeted therapies as adjuvants to conventional hypothermic strategies.

## Introduction

Circulatory arrest is required to provide a bloodless operating field for complex aortic arch surgery. Deep hypothermia can maximize cerebral metabolic and serves as a cornerstone for neuroprotection. Despite this, neurologic injury related to deep hypothermic circulatory arrest (DHCA) remains a significant challenge in contemporary clinical practice. Studies report the incidence of neurologic injury to range from 3.4 to 12% (Montisci et al., 2023). The etiology of brain injury following DHCA is multifaceted, primarily including the duration of circulatory arrest, the rate of rewarming, ischemia/reperfusion (I/R) injury, the pathophysiological state of brain tissue during the profound hypothermia process, and the adjunctive cerebral perfusion techniques (Ziganshin and Elefteriades, 2013; Seese et al., 2023). A recent randomized controlled trial study still found the presence of global cognitive change following DHCA (Hughes et al., 2024; Hughes et al., 2025). It is possible that in clinical practice, relying solely on hypothermia and cerebral perfusion to achieve neuroprotection may be insufficient. There remains a need to explore and uncover the state of brain under conditions of DHCA, to clarify the mechanisms by which DHCA induces injury, and thus to carry out targeted interventional treatments.

Neuroinflammatory responses are implicated in the formation of neurologic impairment/injury following DHCA. It had been found that the inflammatory cytokine levels in the circulation and brain were increased in the DHCA rat model (Yan et al., 2022), and using chlorogenic acid or adiporon can suppress the degree of neuroinflammation via nuclear factor kappa B (NF-κβ) (Chen et al., 2021). In order to elucidate the specific mechanisms underlying the neuroinflammatory response induced by DHCA, proteomics and transcriptomics have begun to be widely applied in the analysis of hippocampal tissue from DHCA rats (Li et al., 2021; Yan et al., 2022; Wang et al., 2023). Our team found that there were 339 circular RNAs (circRNA) dysregulated in the DHCA rat. Among them circFRRS1 was upregulated and validated be RT-Qpcr (Wang et al., 2023). The continued identification of downstream targets of circRNAs will aid in uncovering the mechanisms underlying neurologic injury following DHCA.

Toll-like receptors 4 (TLR4) is a conserved receptor of innate immunity and is expressed across various cell types within the central nervous system, including astrocytes, microglia, and neurons (Sharma et al., 2024). Facing the inflammatory response, the TLR4 is activated along with NF-κβ (Rong et al., 2017). Recent study demonstrated that TLR4/NF-κβ was involved in the process of postoperative cognitive dysfunction after DHCA (Mao et al., 2024), but did not explore upstream regulators. circRNAs are primarily functioning as molecular sponges for microRNAs (miRNAs), thus regulating mRNA expression (Fischer and Leung, 2017). Therefore, we identified circFRRS1 as a novel upstream modulator, bridging non-coding RNA biology with innate immune activation. Our findings would extend previous work implicating TLR4/NF-κB in postoperative cognitive dysfunction.

In this study, we will further investigate the impact of the aforementioned targets on neurological injury after DHCA, building upon previous findings.

## Materials and methods

### Animals

The protocols received institutional review and got approval from the Institutional Animal Care and Use Committee, Fuwai Hospital, Chinese Academy of Medical Sciences (FW-2021-0005). All experimental procedures complied with the Guide for the Care and Use of Laboratory Animals published by the National Institutes of Health. Sprague-Dawley rats were kept under standard laboratory conditions, within free access to food and water (provided by the HFK Bioscience, China). Rats (age, 12–14 weeks; weight, 450–550 g) were randomly allocated into two groups: sham group, DHCA group (*n* = 5, each group).

### DHCA procedure

DHCA procedures were established as previously described (Yan et al., 2022; Yan and Ji, 2022). In the DHCA group, rats were first anesthetized with 2% sevoflurane and then intubated with a 16-G endotracheal tube. Mechanical ventilation was then initiated at a rate of 80 breaths per minute with a tidal volume of 10 mL/kg. Mean arterial blood pressure (MAP) was continuously monitored via the left femoral artery. The tail artery and the right external jugular vein were cannulated and connected to a DHCA circuit, which included a reservoir, a membrane oxygenator, and a heat exchanger. The DHCA circuit was primed with 12 mL of 6% hydroxyethyl starch and 2 mL saline with 150 IU heparin. Cardiopulmonary bypass was then initiated at a flow rate of 160–180 mL/kg/min for 10 min after heparinization (500 IU/kg). Blood flow was directed from the jugular vein through silicon tubes to the membrane oxygenator and then returned to the rat via the tail artery. The target deep temperature was set at 18°C. After 30 min of systemic cooling, DHCA was induced by draining the blood into the reservoir and lasted for approximately 45 min, which was confirmed by MAP = 0. During the rewarming phase, the temperature was gradually increased by rewarming the blood in the DHCA circuits. The rewarming phase lasted for more than 60 min. The rats were then subjected to 40 min for reperfusion to recovery. Afterward, the DHCA circuit was weaned off within another 20 min. Finally, rats were ventilated for 30 min without cardiopulmonary bypass support. Throughout the entire procedure, MAP was maintained above 50 mm Hg. Rats in the sham group were only anesthetized, cannulated, and heparinized.

### Behavioral tests

Before conducting the open field test, animals should be habituated to the testing environment for a brief period to minimize novelty-induced stress. The testing environment consists of a large, square, enclosed area (40 cm × 140 cm) with high walls to prevent escape. On the test day, each animal is placed in the center of the open field arena and allowed to explore the area for 10 min. Video recordings are utilized to collect data on the animals’ movement patterns. DeepLabCut is employed for 2D markerless pose estimation using transfer learning with deep neural networks (Lauer et al., 2022). Motion Sequencing (MoSeq) is an unsupervised machine learning method for analyzing animal behavior (Weinreb et al., 2024). Given behavioral recordings, MoSeq identifies a set of stereotyped movement patterns and their occurrence over time. After extracting movement information with DeepLabCut, a probabilistic time-series model, specifically an autoregressive hidden Markov model (AR-HMM), parses behavior into a set of reusable sub-second motifs known as syllables. This segmentation naturally delineates boundaries between syllables, thereby revealing the structure that governs the interconnections between syllables over time, which we refer to as behavioral grammar.

### Hematoxylin-Eosin and Nissl staining

Hippocampal tissues were immersion fixed in 4% paraformaldehyde and blocked in paraffin. Then they were sectioned at 4 μm. The sections were stained with hematoxylin-eosin (HE) staining (G1004, Servicebio) and Nissl staining (Cresyl Violet; GP2087, Servicebio), respectively.

### Immunohistochemistry

The hippocampal sections were permeabilized with 0.1% Triton X-100 (ZSGB-Bio, Inc.) and incubated with 10% goat serum (ZSGB-Bio, Inc.). Next, the sections were incubated with the primary antibodies in a humidified chamber at 4 °C overnight. The primary antibodies used for immunohistochemistry in this study were NLRP3 (1:200; GB114320, Servicebio) and TLR4 (1:500; GB11519, Servicebio). After rewarming, the sections were incubated with enzyme-conjugated goat anti-rabbit IgG polymer using a Rabbit Two-step kit (ZSGB-Bio, Inc.). Between each step, a phosphate buffered saline (PBS) buffer wash was applied for 5 min each three times. Finally, sections were screened using Pannoramic SCAN (3D HISTECH, Inc.).

### Cell culture and treatment

The PC-12 cells were purchased from Procell Life Science & Technology Co., Ltd. (CL-0481). PC-12 cells were maintained in a PC-12 cell culture medium (CM-0481, Procell) at 37°C in a 95% humidified 5% CO_2_ cell culture incubator. Confluent cultures were passaged by trypsinization. The RNA sequences for transfection were synthesized by GenePharma (Shanghai, China) and listed in Supplementary Table 1. All cell transfections were performed using Lipofectamine 3,000 (Invitrogen; Thermo Fisher Scientific, Inc., Waltham, MA) according to the manufacturer’s protocol. After 24 h transfection, the cells were treated with hypothermia oxygen-glucose deprivation/reperfusion (H-OGD/R) treatment as described before. Briefly, PC-12 cells were placed in a 16°C environment with glucose-free Dulbecco’s modified Eagle’s medium (DMEM) without FBS in an incubator containing 95% N2 and 5% CO_2_ for 2 h. Then the medium was replaced with PC-12 cell culture medium (CM-0481, Procell) and cells were transferred to the incubator set at 37°C with reoxygenation for 24 h. Finally, the cells were harvested for further experiment. CCK-8 assay (G4103, Servicebio) was used for cell proliferation detection.

### Luciferase assays

For luciferase reporter experiments, the specific segments of circFRRS1 and TLR4 predicted to interact with miR-27a-3p were amplified by PCR and inserted into GV272 luciferase reporter vectors (GenePharma Co., Ltd.). The 293T cells were cotransfected in 24-well plates with 0.1 μg of the firefly luciferase report vector and 0.02 μg of the control vector containing Renilla luciferase, pRL-TK (Promega), as well as with 0.4 μg miR-27a-3p mimics, inhibitor or control miRNA. At 48 h post-transfection, luciferase activity was analyzed using the Dual-Luciferase Reporter Assay System (Promega, United States) according to the manufacturer’s instructions. Data were normalized to the Renilla Luminescence and presented relative to control miRNA transfected group.

### Lentiviral infection

The shRNA lentiviral vectors targeting circFRRS1 (sh-circFRRS1) and the negative control (sh-NC) were obtained from Genechem (Shanghai, China). The detailed sequences are listed in Supplementary Table 2. The lentiviruses were administered to the hippocampus of rats using stereotactic injection as previously described (McSweeney and Mao, 2015). Briefly, rats were anesthetized via intraperitoneal injection of sodium pentobarbital (30 mg/kg) and then placed in a stereotaxic apparatus. A hole was drilled at the following coordinates relative to the bregma, according to the rat brain atlas (Swanson, 2018): 3.3 mm posterior, ± 2.0 mm lateral, and 3.0 mm ventral to the skull surface. A 10 μL Hamilton microliter syringe was used to deliver 2 μL of the lentiviral solution (sh-NC or sh-circFRRS1; titer: 1 × 108 TU). Three days after the injection, the rats underwent DHCA surgery. To assess the *in vivo* knockdown efficiency, hippocampal tissues were collected after DHCA intervention, and circFRRS1 expression was detected by RT-qPCR. Rats that did not receive lentivirus treatment were injected with 2 μL of normal saline to control for potential damage caused by the injection procedure itself.

### Western blot

The candidate hippocampal tissues or PC-12 cells used for WB analysis were homogenized and lysed in cold RIPA lysis buffer (Beyotime, Inc.). The protein concentration was measured with BCA assay (Beyotime, Inc.), and an equal amount of protein from all samples was subjected to gel electrophoresis using an 12% sodium dodecyl sulfate-polyacrylamide gel (SDS-PAGE) and then transferred to the nitrocellulose (NC) membranes. NC membranes were blocked with in TBS-T saline (Tris-buffered saline with 0.1% Tween 20) containing 5% non-fat dry milk for 1 h at room temperature. Then, primary antibodies were added and incubated overnight at 4°C. After the primary antibody protocol was completed, mMembranes were then washed in TBST and incubated with secondary antibody at room temperature for 1 h. The primary and secondary antibodies used in this experiment were as follows: primary antibodies: TLR4 (1:1,000; GB11519, Servicebio), Myd88 (1:1,000; GB111554, Servicebio), p-IKK (1:1,000; CST#2697, Cell Signaling Technology), IKK (1:1,000; WL01900, Wanleibio), p-IκB (1:1,000; CST #2859, Cell Signaling Technology), IκB (1:1,000; CST#4812, Cell Signaling Technology), p-P65(1:500; CST#3033, Cell Signaling Technology), P65 (1:1,000; CST#3034, Cell Signaling Technology) and NLRP3 (1:1,000; GB114320, Servicebio); secondary antibodies: HRP conjugated Goat Anti-Rabbit IgG (H+L) (GB23303, Servicebio). Bound proteins were detected using Amersham Imager 800 (Cytiva, Inc.) and quantified by ImageJ software (v1.54).^1^ β-Actin was used as an internal control for protein inputs.

### RT-qPCR

Total RNA was extracted from hippocampal tissues or PC-12 cells with Trizol (Invitrogen, Inc.) following the manufacturer’s protocol, and then converted into cDNA using PrimeScript™ RT reagent kit with gDNA Eraser (Takara Bio, Inc.). RT-qPCR was conducted with SYBR Premix Ex Taq™ II (Takara Bio, Inc.) and the protocol included: pre-denaturation (95 °C, 5 min, 1 cycle) and PCR reaction (95 °C, 10 s, 60 °C, 30 s, 40 cycles in total) followed by a dissolution curve. β-actin and U6 were chosen for the reference genes. The 2^–ΔΔ*CT*^ algorithm was used to calculate the relative quantification expression levels. RT-qPCR primer sequences were listed in Supplementary Table 2.

### Statistical analysis

R (v4.2.1; R Core Team, 2022) was used for data processing and statistical analysis. The experiment and RT-qPCR results were presented as mean ± SD. Student’s *t*-tests were used for comparisons between the two groups. One-way ANOVA with post hoc analysis was applied for all multi-group comparison. *P* < 0.05 were considered to be statistically significant.

## Results

**FIGURE 1:**
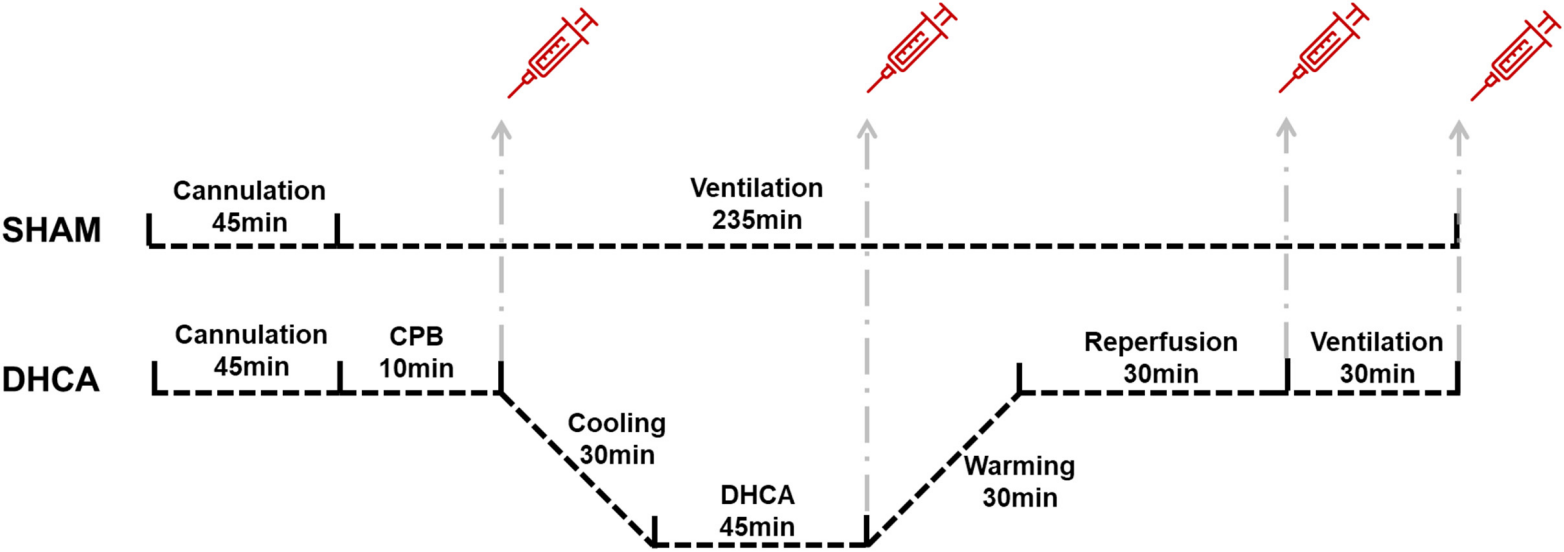
The protocol of the whole DHCA experiment. CPB, cardiopulmonary bypass; DHCA, deep hypothermic circulatory arrest.

The protocol of the whole DHCA experiment was presented in Figure 1. Total 10 rats were randomly divided into two groups (5 for sham group and 5 for DHCA group). Blood gas analysis in rats of each group was listed in Table 1.

**TABLE 1:** Blood gas parameters of the rats.

	CPB 10 min		DHCA 45 min		Reperfusion 60 min		Ventilation 30 min	
Parameters	SHAM	DHCA	SHAM	DHCA	SHAM	DHCA	SHAM	DHCA
Hb (g/L)	11.34	5.63^c^	11.64	6.58^c^	11.26	7.53^c^	11.50	8.93**^b^**
Hct (%)	33.40	16.00**^c^**	34.20	19.00**^c^**	33.20	22.00**^c^**	33.80	26.25**^b^**
pH	7.41	7.49	7.39	7.69^a^	7.41	7.36	7.41	7.21^a^
PaO_2_ (mm/Hg)	524.40	492.50	491.80	718.50^c^	458.00	467.80	460.60	278.00^b^
PaCO_2_ (mm/Hg)	50.24	39.40^a^	55.46	21.03^c^	53.28	27.90^c^	53.14	48.10
Lac (mmol/L)	0.53	0.63	1.15	5.00^c^	1.51	7.30^a^	1.90	6.37^a^
HCO_3_^–^ (mmol/L)	31.86	30.10	33.56	23.90^a^	33.20	16.88^c^	33.82	19.33^c^
Na^+^ (mmol/L)	140.80	143.80	139.60	139.50	138.80	138.00	137.80	141.00
K^+^ (mmol/L)	3.50	3.13	3.96	3.15^b^	3.74	4.48	4.02	4.80
Cl^–^ (mmol/L)	103.60	115.30	102.00	109.50^a^	101.80	108.00^a^	100.00	110.80^b^
BUN (mmol/L)	11.80	10.50	13.80	14.25	13.60	17.25	15.20	21.75
GLU (mmol/L)	181.40	169.00^c^	171.60	352.30^c^	154.20	495.00^c^	157.80	410.30^c^
SaO_2_ (%)	100	100	100	100	100	100	100	100

CPB, cardiopulmonary bypass; DHCA, deep hypothermia circulatory arrest; Hb, hemoglobin; Hct, hematocrit; Lac, lactate; PaO_2_, partial pressure of arterial oxygen; PaCO_2_, partial pressure of arterial carbon dioxide. Lac, lactate; BUN, blood urea nitrogen; GLU, glutamic acid.

^a^*p* < 0.001 vs. Sham.

^b^*p* < 0.01 vs. Sham.

^c^*p* < 0.05 vs. Sham.

### DHCA induces delirium-like behavior in rats

**FIGURE 2:**
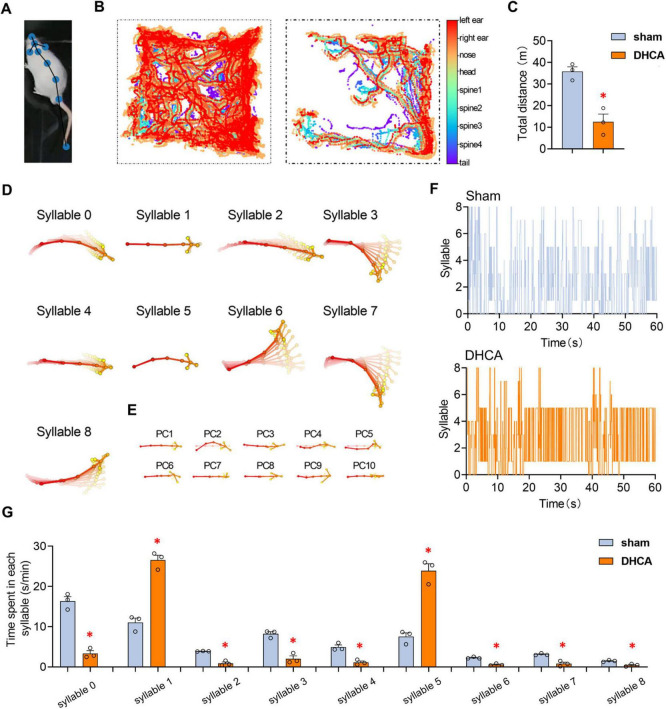
DHCA induces delirium-like behavior in rats. **(A)** Rat skeleton illustrating the nose, left ear, right ear, head, body (spine segments 1–4), and tail for pose estimation in DeepLabCut. **(B)** Trajectory of rat movement during the open field test. **(C)** DHCA rats exhibited a decreased total traveling distance in the open field test. **(D)** Plots showing the median trajectory of poses associated with specific syllables: working (syllables 0, 4, 8), left or right turning (syllables 6, 7), freezing (syllables 1 and 5), right looking (syllable 3), and running (syllable 2). **(E)** Aligned and centered keypoint coordinates fitted during motion sequencing. **(F)** Representative syllable transitions of Sham and DHCA rats. **(G)** Statistics on the average time spent in each syllable per minute during the open field test for Sham and DHCA rats. DHCA, deep hypothermic circulatory arrest. *p < 0.05, vs. Sham.

Postoperative cognitive dysfunction encompasses delirium. However, in rats, cognitive status can only be indirectly assessed through their physical behaviors. Therefore, to validate the behavioral changes in DHCA rats, we assessed their performance in the open field test and marked their poses and movement trajectories using DeepLabCut (Figures 2A,B). Notably, rats subjected to DHCA exhibited a significant decrease in total traveling distance compared to the control group (Sham rats) (Figure 2C), suggesting impairment in exploratory behavior indicative of delirium-like symptoms (Cao et al., 2023). Further motion sequencing (Moseq) analysis revealed distinct patterns in the median trajectory of poses associated with specific movement syllables. The behaviors identified included working (syllables 0, 4, 8), left or right turning (syllables 6, 7), freezing (syllables 1 and 5), right looking (syllable 3), and running (syllable 2) (Figure 2D). Keypoint analysis provided aligned and centered coordinates during motion sequencing, unveiling a clear pattern of behavioral transitions (Figure 2E). The comparison of syllable transitions between Sham and DHCA rats illustrated distinct differences (Figure 2F). Importantly, we quantified the average time spent in each syllable per minute during the open field test, revealing that DHCA rats spent significantly more time in freezing behaviors and less time in exploration compared to the Sham group (Figure 2G), indicative of heightened anxiety or confusion. These findings collectively provide compelling evidence that DHCA results in significant behavioral changes in rats, reflecting symptoms consistent with delirium.

### DHCA induces significant hippocampal damage

**FIGURE 3:**
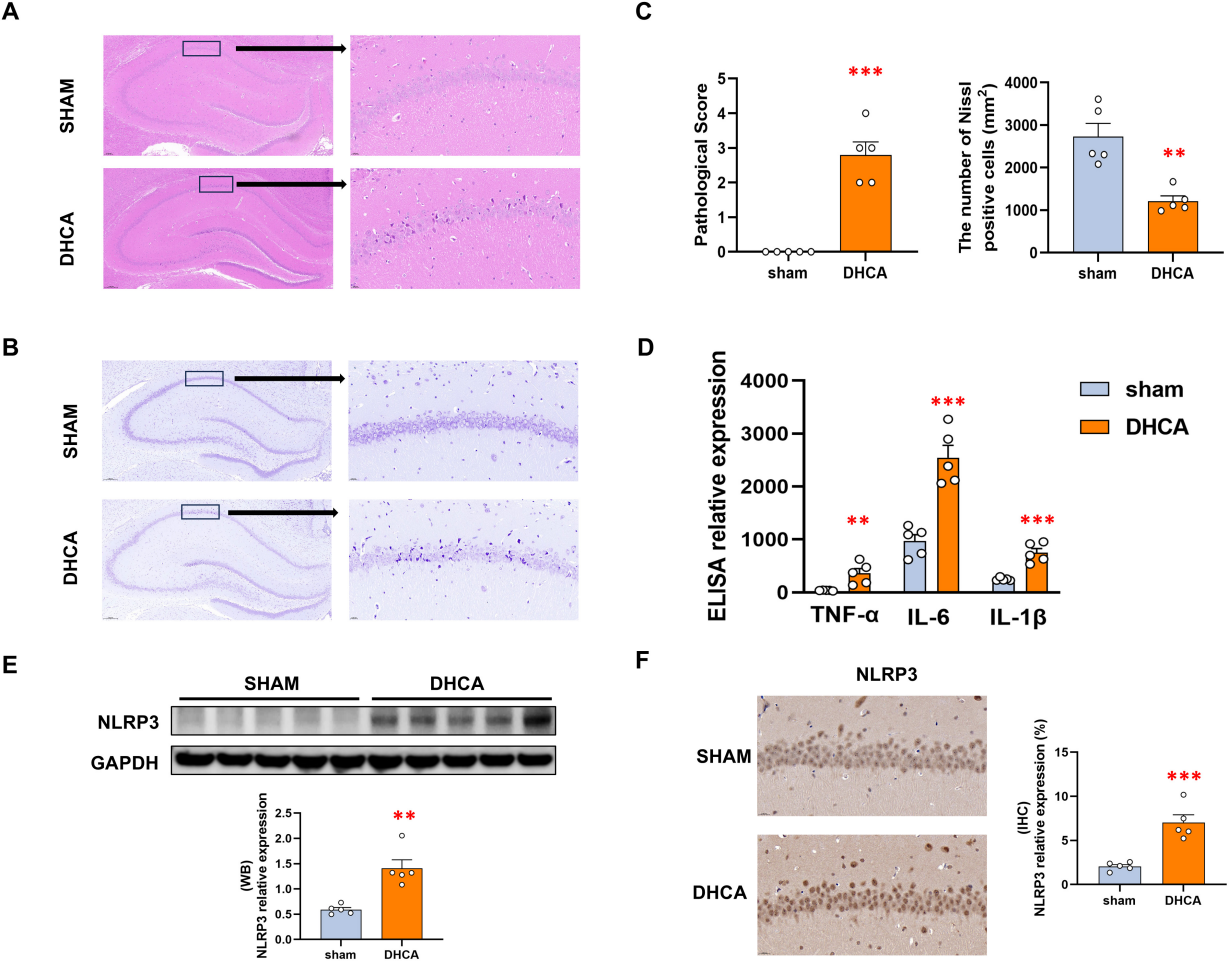
DHCA induces significant hippocampal damage and inflammation activation. **(A)** Representative HE staining revealed higher numbers of necrotic neurons in the hippocampus regions of DHCA group than the Sham group (Enlarged region: CA1). **(B)** Representative Nissl staining showed shrunken and deeply stained neurons and the disappeared Nissl body in the hippocampus of DHCA group (Enlarged region: CA1). **(C)** Statistical analysis for HE and Nissl staining results. **(D)** ELISA analysis showed the higher hippocampus levels of TNF-α, IL-6, and IL-1β in the DHCA group. **(E,F)** Both WB and immunohistochemistry confirmed NLRP3 inflammasome activation in the DHCA hippocampal tissue. DHCA: deep hypothermic circulatory arrest. **p < 0.01, ***p < 0.001, vs. Sham.

The HE staining revealed remarkable neuronal abnormalities in the hippocampus of rats in the DHCA group, evidenced by high numbers of necrotic neurons (Figures 3A,C). The Nissl staining also showed marked neuronal changes in the hippocampus of rats in the DHCA group, including the shrunken and deeply stained cell bodies and the disappeared Nissl body (Figures 3B,C). ELISA results showed that DHCA induced increasing levels of inflammatory factor (Figure 3D). Further WB analysis showed that the expression of NLRP3 was elevated in the DHCA group (Figure 3E). Immunohistochemistry staining of NLRP3 also displayed a similar trend to the WB results (Figure 3F). These results revealed that DHCA cause severe inflammation damage in hippocampal tissue.

### CircFRRS1 is a novel target to inhibit DHCA-induced hippocampal damage

**FIGURE 4:**
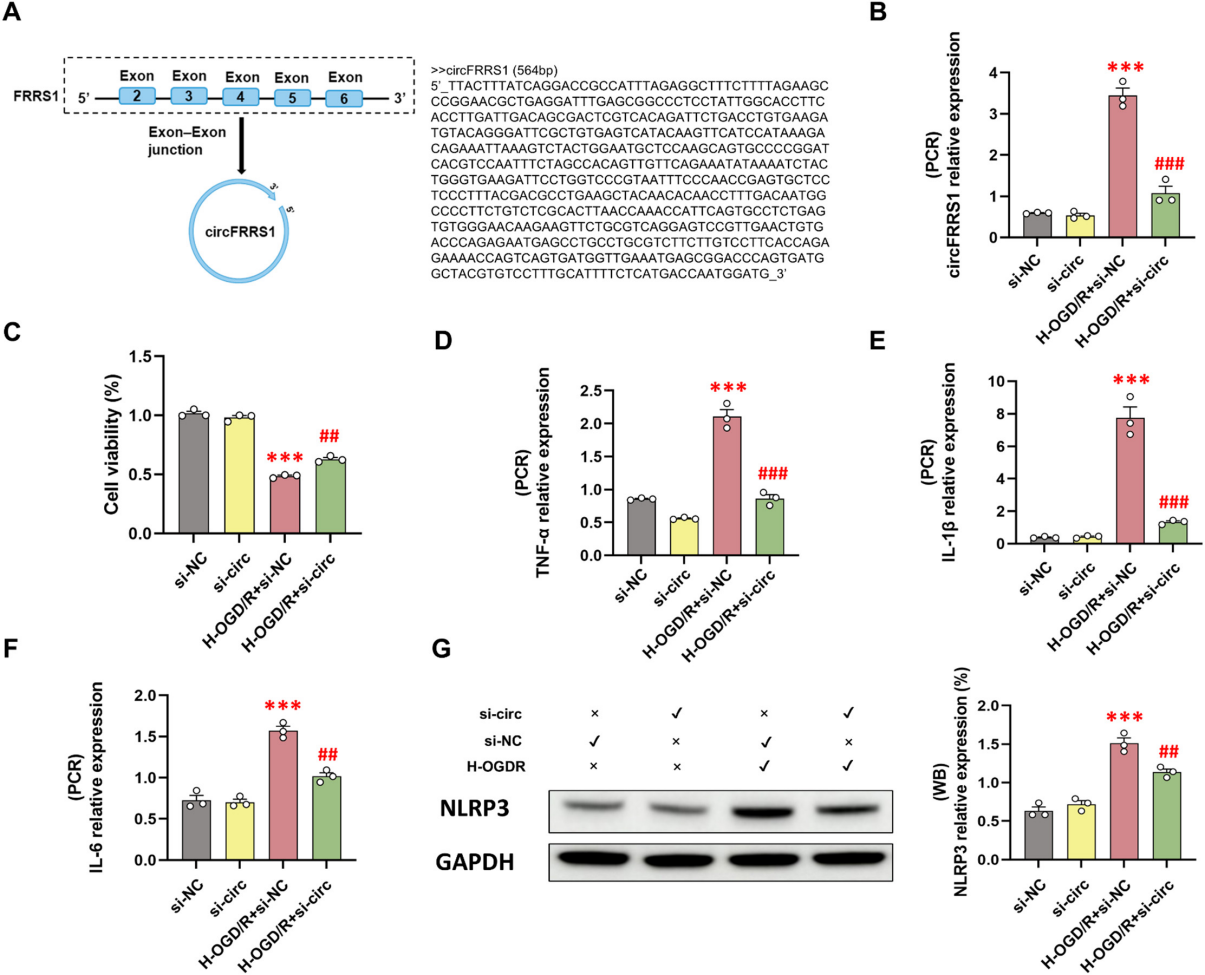
Inhibition of circFRRS1 alleviates the inflammation damage of PC-12 cells after H-OGD/R treatment. **(A)** Sequence diagram of circFRRS1. **(B)** The inhibition effects of si-circFRRS1 were detected by RT-PCR. **(C)** CCK8 assay revealed circFRRS1 inhibition improved the survival rate of H-OGD/R treated PC-12 cells. **(D–F)** The inhibition of circFRRS1 reduced the increasing level of TNF-α, IL-6, and IL-1β induced by H-OGD/R treatment. **(G)** The inhibition of circFRRS1 alleviated the NLRP3 inflammasome activation of H-OGD/R treated PC-12 cells. ***p < 0.001, vs. si-NC group; ^##^*p* < 0.01, ^###^*p* < 0.001, vs. H-OGD/R + si-NC group.

In the previous study we have analyzed the expression profiles of circRNAs in rat hippocampus after DCHA. Among all dysregulated circRNAs, rno_circ_0028462 (rename as rno-circFRRS1, due to the host gene was FRRS1) confirmed highly expressed in DHCA group with both transcripts analysis and experiment validation (Wang et al., 2023). The detail sequence of circFRRS1 was shown in Figure 4A. In this study, we further explored the downstream mechanism of circFRRS1. *In vitro* experiments, PC-12 cells were treated with H-OGD/R to simulate DHCA process and transfected with si-circFRRS1. As shown in Figure 4B, the transfection of si-crcFRRS1 markedly reduced the high level of circFRRS1 induced by H-OGD/R in PC-12 cells. CCK-8 assays revealed that suppressing circFRRS1 raised the survival rate of PC-12 cells treated with H-OGD/R (Figure 4C). In addition, silencing circFRRS1 inhibit the inflammatory activation by H-OGDR, evidenced by decreased gene expression of TNF-α, IL-6, and IL-1β and less NLRP3 protein (Figures 4D–G).

### miR-27a-3p/TLR4 axis serves as the downstream target of circFRRS1

**FIGURE 5:**
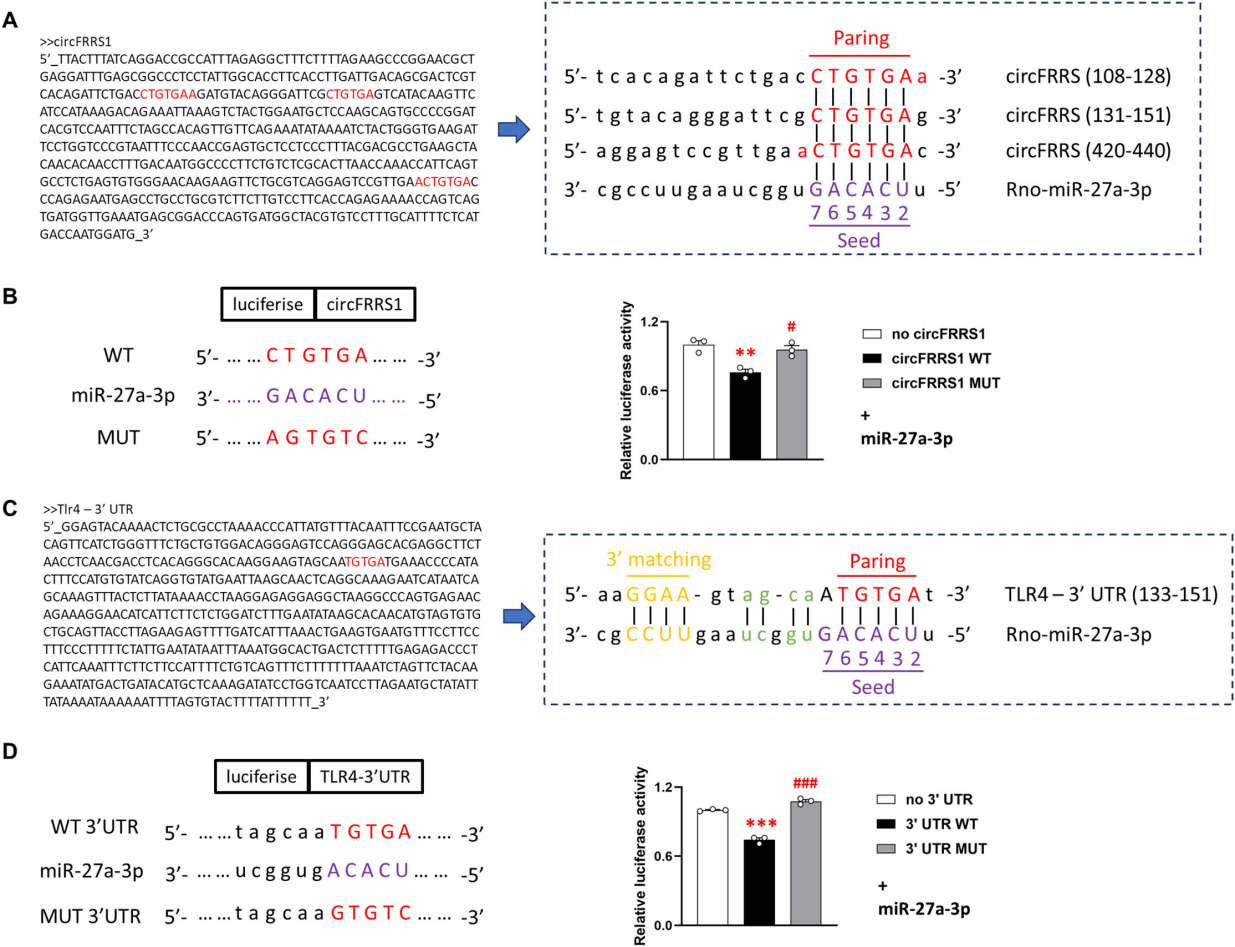
Dual luciferase reporter experiment confirmed the regulatory relationships of circFRRS1/rno-miR-27a-3p and rno-miR-27a-3p/TLR4 pairs. **(A)** The predicting binding site between circFRRS1 and miR-27a-3p. **(B)** Dual luciferase reporter assay revealed that miR-27a-3p attenuated the luciferase activity of circFRRS1-WT, but not of circFRRS1-MUT. **(C)** The predicting binding site between TLR4 and miR-27a-3p. **(D)** Dual luciferase reporter assay revealed that miR-27a-3p attenuated the luciferase activity of TLR4 3’-WT, but not of TLR4 3’-MUT. **p < 0.01, ***p < 0.001, vs. control group; ^#^*p* < 0.05, ^###^*p* < 0.001, vs. WT group.

A key function of circRNA is to sequester miRNA by sponging, resulting in alteration of mRNA targets. In our previous study, bioinformatics analysis showed that miR-27a-3p was a candidate for circFRRS1 (Wang et al., 2023). Using miRTarbase, which display the largest amount of validated miRNA/mRNA pairs (Huang et al., 2020), the rno-miR-27a-3p/TLR4 pair was shown as strong evidence (Supplementary Figure 1), and miRWalk database showed that the interaction between miR-27a-3p and TLR4 was predicted across species (Supplementary Figure 2). Recent studies have shown that miR-27a-3p/TLR4 pair was a novel miRNA-related target that plays a major role in inflammatory activation and I/R injury, thus miR-27a-3p/TLR4 pair was selected as one candidate (Wang et al., 2019; Luo et al., 2022). In order to confirm the bioinformatics result, the dual luciferase reporter assay was performed and the regulatory relationships of circFRRS1/rno-miR-27a-3p and rno-miR-27a-3p/TLR4 pairs were confirmed (Figure 5).

### DHCA induces downregulation of miR-27a-3p expression and upregulation of TLR4 axis in hippocampal tissues

**FIGURE 6:**
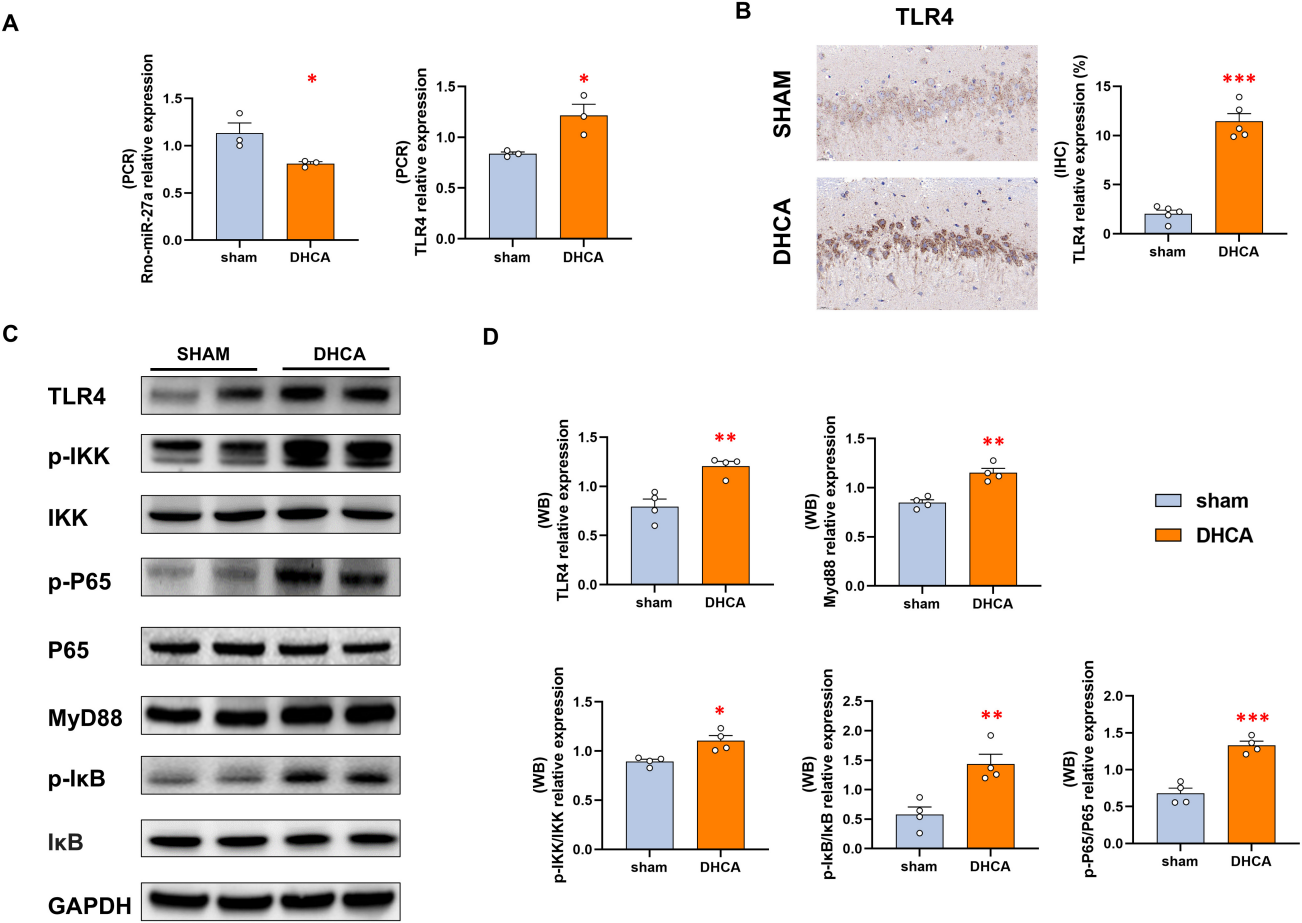
DHCA induces abnormal changes of miR-27a-3p/TLR4 pair and downstream axis. **(A)** The RT-qPCR analysis showed the lower expression level of miR-27a-3p and higher level of TLR4 in the DHCA hippocampus tissues. **(B)** Immunohistochemistry confirmed that the TLR4 expression was significantly increased in the DHCA rat hippocampus. **(C)** WB analysis showed that the key proteins of TLR4/NF-κB/NLRP3 signaling were activated in the DHCA rat hippocampus tissues. **(D)** Statistical analysis of WB results. DHCA, deep hypothermic circulatory arrest. *p < 0.05; **p < 0.01, ***p < 0.001, vs. Sham.

Next, the expression tendency of miR-27a-3p/TLR4 pair was validated in the rat hippocampus tissues. The RT-PCR analysis results showed that the lower expression level of miR-27a-3p and higher level of TLR4 in the DHCA hippocampus tissues (Figure 6A). Immunohistochemistry showed that TLR4 was mainly expressed on the cell membrane, and confirmed that its expression was significantly increased in the DHCA group compared to the sham rats at the protein level (Figure 6B). TLR4/NF-κB signaling is an important pathway that triggers the formation of NLRP3 inflammasome, thus we detected the expression levels of key proteins in this pathway. As shown in Figures 6C,D, levels of TLR4, MyD88, and the ratios of p-IKK/IKK, p-IκB/IκB, and p-P65/P65 were markedly elevated in DHCA rat hippocampus relative to the Sham group.

### The inhibition of circFRRS1 reduces TLR4 mediated inflammatory response in H-OGD/R-induced PC-12 cells

**FIGURE 7:**
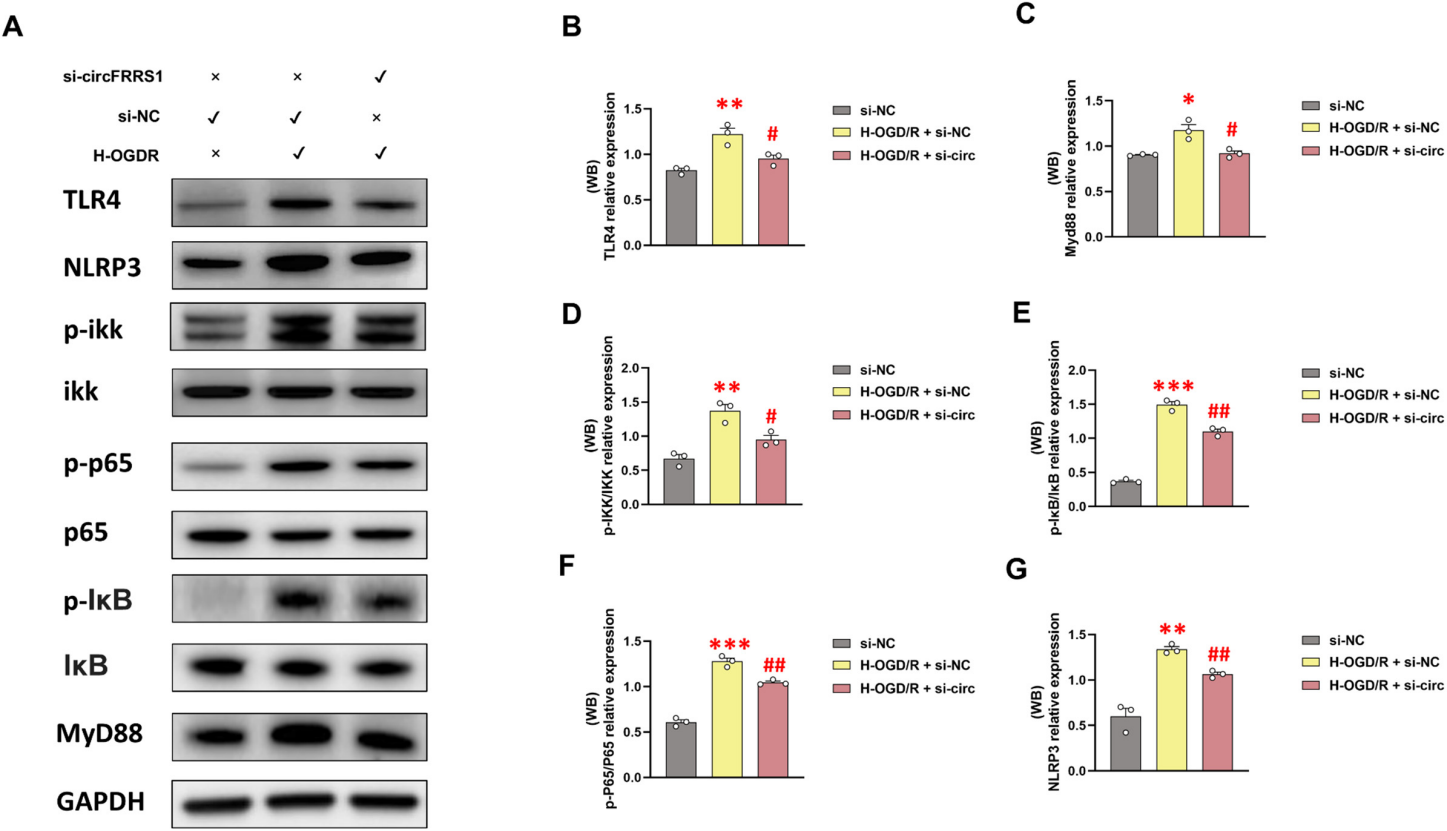
The inhibition of circFRRS1 suppressed TLR4/NF-κB/NLRP3 pathway in H-OGD/R-induced PC-12 cells. **(A)** WB analysis revealed that the TLR4/NF-κB/NLRP3 axis was activated in H-OGD/R-induced PC-12 cells and markedly inhibited after si-circFRRS1 transfected. **(B–G)** Statistical analysis of WB results. *p < 0.05; **p < 0.01, ***p < 0.001, vs. si-NC group; ^#^*p* < 0.05, ^##^*p* < 0.01, vs. H-OGD/R + si-NC group.

We further used WB analysis to evaluate whether inhibition of circFRRS1 suppressed NLRP3 inflammasome activation by regulating the TLR4/NF-κB axis in H-OGD/R-treated PC-12 cells. As shown in Figure 7, levels of TLR4, MyD88, NLRP3, and the ratios of p-IKK/IKK, p-IκB/IκB, and p-P65/P65 were markedly elevated after H-OGD/R treatment relative to the controls. Levels of these proteins were markedly reduced in H-OGD/R + si-circFRRS1-treated cells compared with those treated with H-OGD/R + si-NC cells.

### The regulation of TLR4 by circFRRS1 is mediated through miR-27a-3p

**FIGURE 8:**
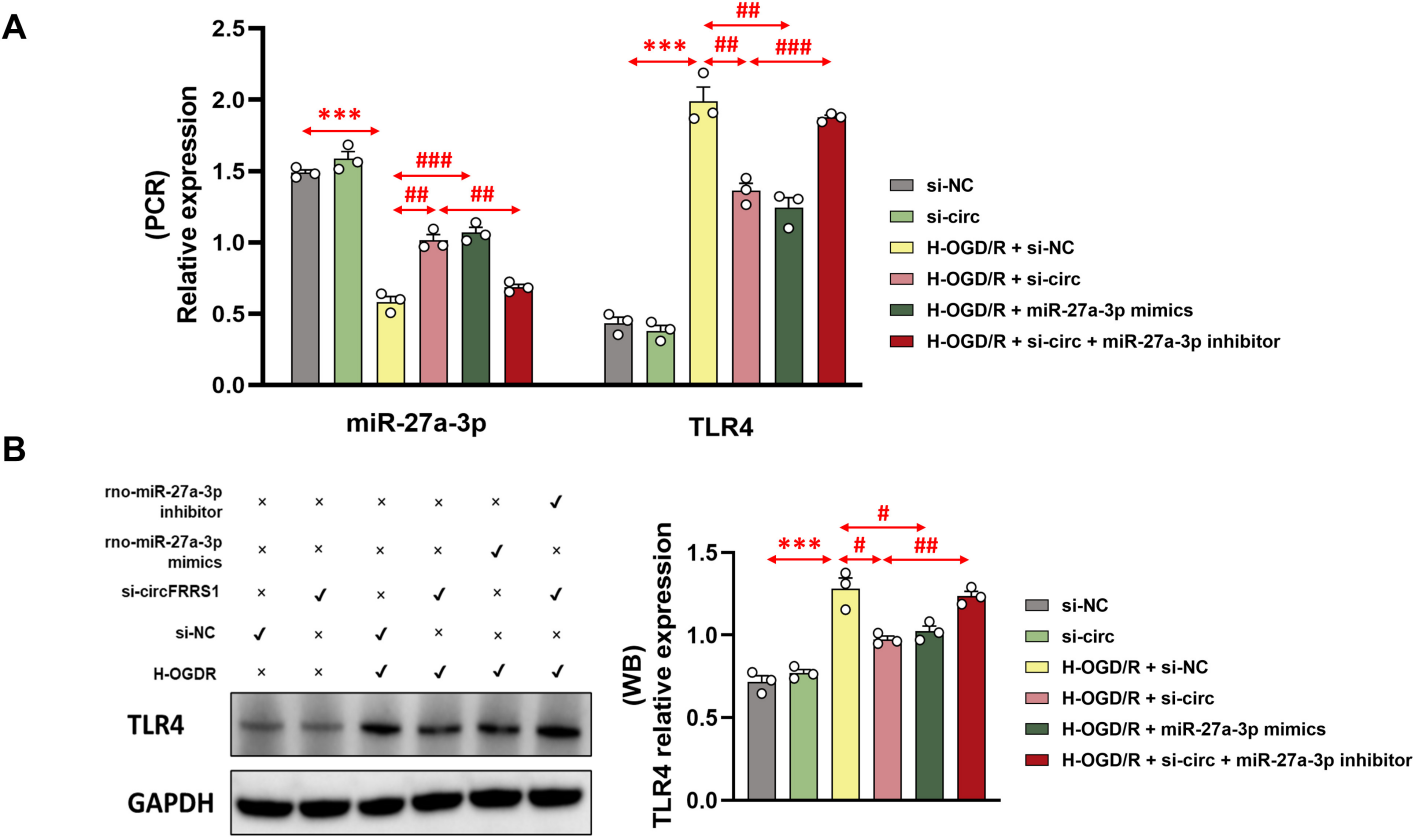
circFRRS1 can regulate TLR4 by targeting miR-27a-3p. **(A)** Administration of miR-27a-3p inhibitor could reverse the downregulation of TLR4 expression by transfecting si-circFRRS1. **(B)** Further protein-level detection using WB gave the further validation. ***p < 0.001, vs. si-NC group; ^#^*p* < 0.05, ^##^*p* < 0.01, ^###^*p* < 0.001, vs. H-OGD/R + si-NC group or H-OGD/R + si-circ group.

To confirm that the regulation of TLR4 by circFRRS1 in H-OGD/R-treated PC-12 cells is mediated by miR-27a-3p, we performed additional cell experiments. As shown in Figure 8A, administration of miR-27a-3p inhibitor could reverse the downregulation of TLR4 expression by inhibiting circFRRS1. Further protein-level detection gave the same results (Figure 8B).

### Reduced expression of circFRRS1 alleviates hippocampal damage in DHCA rats by regulating the miR-27a-3p/TLR4 pathway

Furthermore, we administered shRNA lentiviral vectors targeting circFRRS1 (sh-circFRRS1) to rats subjected to DHCA in advance (Supplementary Figure 3A). The vivo knockdown efficiency of circFRRS1 was assessed through RT-qPCR (Supplementary Figure 3B). The level of miR-27a-3p was significantly increased in the DHCA +sh-circFRRS1 group compared to the DHCA + sh-NC group. In DHCA rats, sh-circFRRS1 intervention restored the expression of miR-27a-3p and suppressed TLR4 protein levels in hippocampal tissue (Supplementary Figure 3C). Additionally, the DHCA + sh-circFRRS1 group exhibited lower NLRP3 protein expression compared to the DHCA + sh-NC group, indicating reduced inflammatory activation (Supplementary Figure 3C). Histopathological examination, including HE and PAS staining, revealed less damage in the hippocampal tissue of the DHCA + sh-circFRRS1 group relative to the DHCA + sh-NC group (Supplementary Figures 3D,E). In conclusion, the above results demonstrated that inhibition of circFRRS1 effectively alleviated inflammatory damage in the hippocampal tissue, further supporting the potential of circFRRS1 as an important therapeutic target for DHCA-associated neurological injury.

## Discussion

**FIGURE 9:**
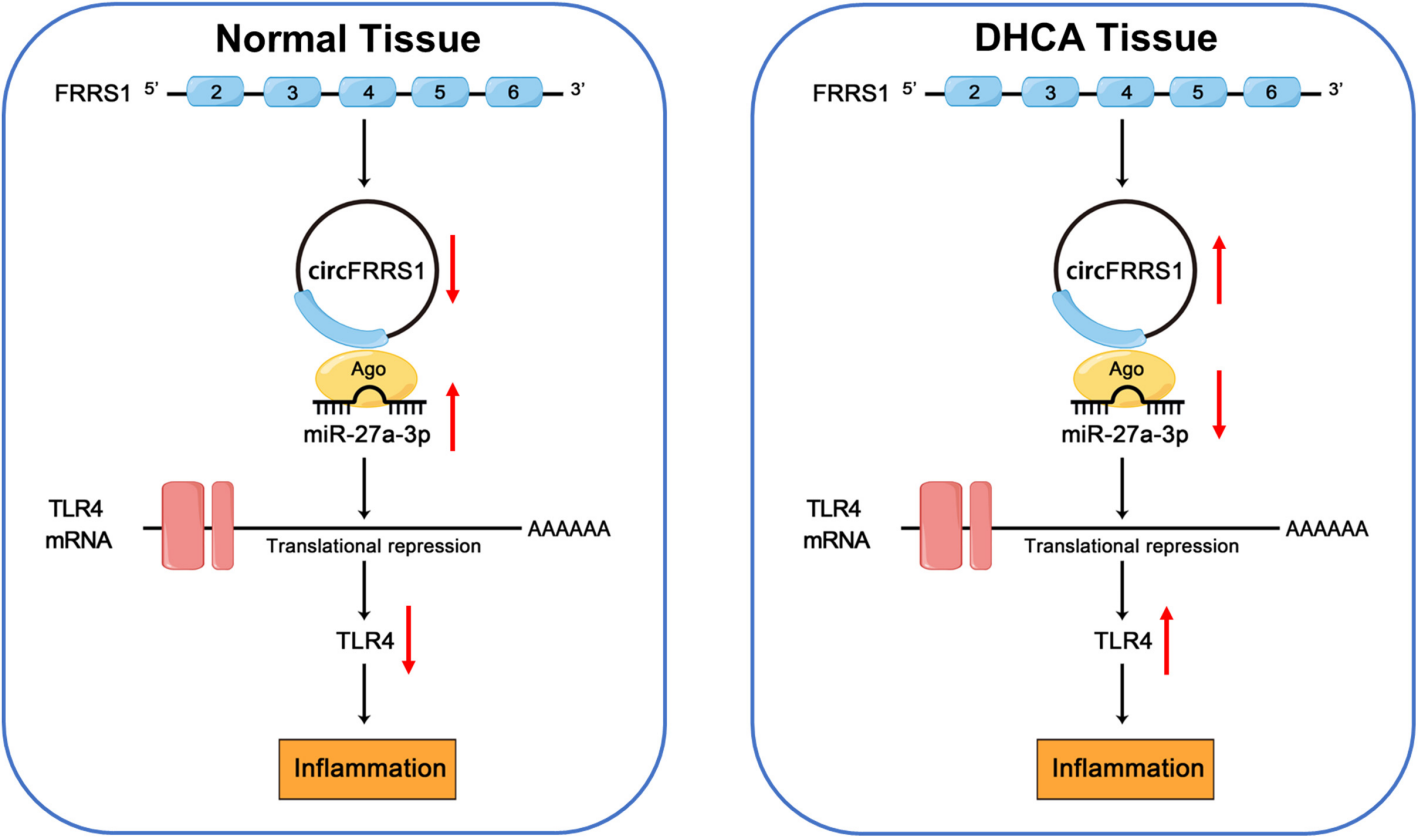
The central picture of the whole study. DHCA, deep hypothermic circulatory arrest.

Neurological cognitive impairment following DHCA is a common complication, though the precise mechanisms driving its development remain incompletely understood. Our research team has long focused on this area of investigation. This study demonstrates that DHCA induces delirium-like behavioral deficits and hippocampal neuroinflammation in rats, mediated through the circFRRS1/miR-27a-3p/TLR4/NF-κB axis (Figure 9). This work provides the first evidence linking circRNA-mediated epigenetic regulation to TLR4-driven neuroinflammation in the context of DHCA, revealing a previously unrecognized mechanistic pathway contributing to neurological injury.

Since the introduction of DHCA in the 1950s, the significance of post-procedural neurological injury has become increasingly recognized. Some researchers attribute these complications to the inherent effects of deep hypothermia itself, which has spurred clinical exploration of alternative strategies, such as moderate hypothermic or even normothermic circulatory arrest (Abdulwahab et al., 2024; Zhang K. et al., 2024; Rings et al., 2025). Others propose that insufficient cerebral perfusion during the arrest period leads to ischemia-reperfusion (I/R) injury, a theory that has driven the adoption of adjuncts like antegrade cerebral perfusion (ACP) or retrograde cerebral perfusion (RCP) into surgical practice (Ede et al., 2024; Shah et al., 2025). While these interventions have demonstrated partial therapeutic efficacy, current clinical approaches and related research have yet to conclusively identify the precise mechanisms underlying DHCA-induced neurological damage. Continued animal studies remain essential to elucidate potential pathogenic pathways, which may inform more effective strategies for neuroprotection across diverse clinical settings. Accordingly, our team continues to utilize DHCA animal models to systematically evaluate the neurological impacts of critical procedural components, including profound hypothermia, circulatory arrest, and rewarming processes (Liu et al., 2019a; Liu et al., 2019b; Liu et al., 2020; Yan et al., 2022; Yan and Ji, 2022; Wang et al., 2023).

The postoperative cognitive changes are induced by structural and functional alterations in hippocampal tissue following DHCA. As early as 2021, studies investigated the expression profiles of circRNAs in hippocampal tissue after DHCA (Li et al., 2021). Our research team subsequently conducted replication experiments and identified a significant upregulation of circ_0028462 (circFRRS1) expression following DHCA (Wang et al., 2023). These findings have laid the scientific foundation for our current research.

Although DHCA mitigates neurological injury caused by I/R following circulatory arrest through deep hypothermia, our rat model still demonstrated widespread inflammatory activation within the hippocampus. Therefore, identifying molecular pathways activated by I/R injury and intervening accordingly may further reduce neurological complications associated with DHCA. The TLR4/NF-κB pathway is a well-established pro-inflammatory signaling cascade, and targeted intervention of this pathway has been extensively explored and translated in various disease models of I/R-induced neurological injury (Cai et al., 2024; Zhang L. et al., 2024). Non-coding RNAs have recently been identified as innovative molecular targets for therapeutic intervention. In contrast to traditional pharmacological strategies, non-coding RNAs provide the potential for precise modulation of specific gene expression (Nappi, 2024). Previous studies have shown that downregulation of miR-27a-3p promotes TLR4/NF-κB activation and plays a critical role in inflammation-related injury across multiple organs (Luo et al., 2022; Wang et al., 2025). In this study, we indirectly confirm the key role of the miR-27a-3p/TLR4/NF-κB axis in the regulation of I/R-induced neuroinflammation using DHCA rat model, and further identify a novel upstream regulator—circFRRS1. These results enrich the understanding of I/R injury and provide new insights for therapeutic intervention and clinical translation.

In addition, our findings extend previous work implicating TLR4/NF-κB in postoperative cognitive dysfunction. For instance, Mao and Tang et al. reported TLR4 upregulation in DHCA models but did not explore upstream regulators (Mao et al., 2024). By contrast, we identified circFRRS1 as a novel upstream modulator, bridging non-coding RNA biology with innate immune activation. Similarly, studies have reported that miR-27a-3p can induce M1 polarization of macrophages (Zhao et al., 2023). This insight suggests that elevated miR-27a-3p levels in central nervous system microglia may promote their M1-type polarization, thereby manifesting pro-inflammatory functions. Meanwhile, studies have also found that miR-27a-3p plays a pro-inflammatory role in the pathophysiology of infectious endophthalmitis (Chen et al., 2024). In a rat model of intracerebral hemorrhage (ICH), downregulation of miR-27a-3p exacerbates cerebral edema, disrupts blood-brain barrier (BBB) integrity, and aggravates neurological injury (Xi et al., 2018). miR-27a-3p also plays a role in the hypoxia-induced cardiomyocyte injuries (Lei et al., 2020). Its role in DHCA-associated neuroinflammation is newly established here. Notably, our use of unsupervised machine learning (MoSeq) to quantify delirium-like behavior represents a methodological advance over traditional scoring systems, enhancing objectivity in assessing neurological outcomes.

Several limitations must be acknowledged. First of all, the proposed molecular mechanism, in which circFRRS1 regulates TLR4 expression by sponging miR-27a-3p, is primarily supported by evidence from dual-luciferase reporter assays. While these results are indicative, direct evidence for the endogenous binding between circFRRS1 and miR-27a-3p within cells—such as validation through RNA Immunoprecipitation or circRNA pull-down assays—is currently lacking. This gap may impose constraints on the completeness of the mechanistic interpretation. Future studies will aim to incorporate these experimental approaches to provide more direct and comprehensive insights into the precise role of circFRRS1 within the relevant signaling pathway. Second, this study focused on hippocampal tissue, while other brain regions (e.g., cortex) may contribute to behavioral deficits. Meanwhile, PC-12 cells, while widely used, lack the complexity of primary neurons or glial cells. Finally, *in vivo* silencing of circFRRS1 was not performed; thus, the therapeutic potential of targeting this axis remains speculative. Future studies will also validate these findings in larger cohorts and explore circFRRS1 inhibition *in vivo*. Investigating crosstalk between circFRRS1 and other neuroinflammatory pathways (e.g., complement system, microglial activation) could provide a more comprehensive understanding. Translational efforts might include developing circRNA-targeted therapies or combining miR-27a-3p mimics with existing neuroprotective strategies. Additionally, multi-omics approaches (e.g., single-cell RNA-seq) could resolve cell-type-specific responses to DHCA, refining therapeutic targeting.

In conclusion, we identified circFRRS1 as a novel regulatory RNA that exacerbates DHCA-induced neuronal injury by acting as a sponge for miR-27a-3p, which in turn relieves repression of TLR4 and activates downstream NF-κB signaling and NLRP3 inflammasome assembly. This study unveils a circRNA-dependent mechanism driving DHCA-associated neuroinflammation, offering new avenues for intervention beyond conventional hypothermic strategies. By integrating behavioral, molecular, and computational approaches, we provide a framework for understanding and mitigating neurologic injury in complex aortic surgery.

## Data Availability

The original contributions presented in the study are included in the article/Supplementary material, further inquiries can be directed to the corresponding authors.

## Footnotes

1
http://imagej.net/ImageJ
