# Supplementary material for: CircFRRS1 drives neuroinflammation through the miR-27a-3p/TLR4 pathway after deep hypothermic circulatory arrest
Source: Front Cell Neurosci. 2026 Feb 25;20:1750887. doi: 10.3389/fncel.2026.1750887 (PMC12975488; doi:10.3389/fncel.2026.1750887)
Supplement: Supplementary file 1 [file Data_Sheet_1.pdf]

Supplementary Table 1: The RNA sequences for transfection.

Term	Sequence
si-NC	Sense (5'-3')<br>UUCUCCGAACGUGUCACGUTT<br>Antisense (5'-3')<br>ACGUGACACGUUCGGAGAATT
si-circFRRS1	Sense (5'-3')<br>GACCAAUGGAUGUUACUUUTT<br>Antisense (5'-3')<br>AAAGUAACAUCCAUUGGUCTT
rno-miR-27a-3p mimics	Sense (5'-3')<br>UUCACAGUGGCUAAGUUCCGC<br>Antisense (5'-3')<br>GGAACUUAGCCACUGUGAAUU
rno-miR-27a-3p inhibitor	GCGGAACUUAGCCACUGUGAA

Supplementary Table 2: shRNA sequences.

Term	Primer sequence
sh-NC	TTCTCCGAACGTGTCACGT
sh-circFRRS1	ACCAATGGATGTTACTTTA

Supplementary Table 3: RT-qPCR primer sequences.

Primer	Sense (5'-3')	Antisense (5'-3')
circFRRS1	GAAATGAGCGGACCCAGTGA	CGCTCAAATCCTCAGCGTTC
GAPDH	AAGTTCAACGGCACAGTCAAG	TACTCAGCACCAGCATCACC
rno-TLR4	ATGAGGACTGGGTGAGAAAC	ACCAACGGCTCTGGATAAAG
rno-TNF-α	GATCGGTCCCAACAAGGAGG	CTTGGTGGTTTGCTACGACG
rno-IL-6	ACAAGTCCGGAGAGGAGACT	GAATTGCCATTGCACAACTCT
rno-IL-1β	CAGCTTTCGACAGTGAGGAGA	TGTCGAGATGCTGCTGTGAG
rno-miR-27a-3p	TTCACAGTGGCTAAGTTCCGC	GCAGGGTCCGAGGTATTC
U6	CTCGCTTCGGCAGCACA	AACGCTTCACGAATTTGCGT

Page 1 of 1 | 1

ID	Species (miRNA)	Species (Target)	miRNA	Target	Validation methods: Strong evidence			Validation methods: Less strong evidence				Sum	# of papers
					Reporter assay	Western blot	qPCR	Microarray	NGS	pSILAC	Other		
MIRT001102	Rattus norvegicus	Rattus norvegicus	rno-miR-27a-3p	Rxra	✔	✔	✔				✔	4	1
MIRT002211	Rattus norvegicus	Rattus norvegicus	rno-miR-27a-3p	Hyou1				✔			✔	2	1
MIRT004512	Rattus norvegicus	Rattus norvegicus	rno-miR-27a-3p	Trim63	✔						✔	2	1
MIRT005428	Rattus norvegicus	Rattus norvegicus	rno-miR-27a-3p	Thrb	✔	✔					✔	3	1
MIRT731854	Rattus norvegicus	Rattus norvegicus	rno-miR-27a-3p	Foxo3	✔	✔	✔					3	1
MIRT732190	Rattus norvegicus	Rattus norvegicus	rno-miR-27a-3p	Irak4	✔	✔	✔					3	1
MIRT732191	Rattus norvegicus	Rattus norvegicus	rno-miR-27a-3p	Tlr4	✔	✔	✔					3	1

Page 1 of 1 | 1

Supplementary Figure 1: Results of rno-miR-27a-3p target genes in miRTarget database.

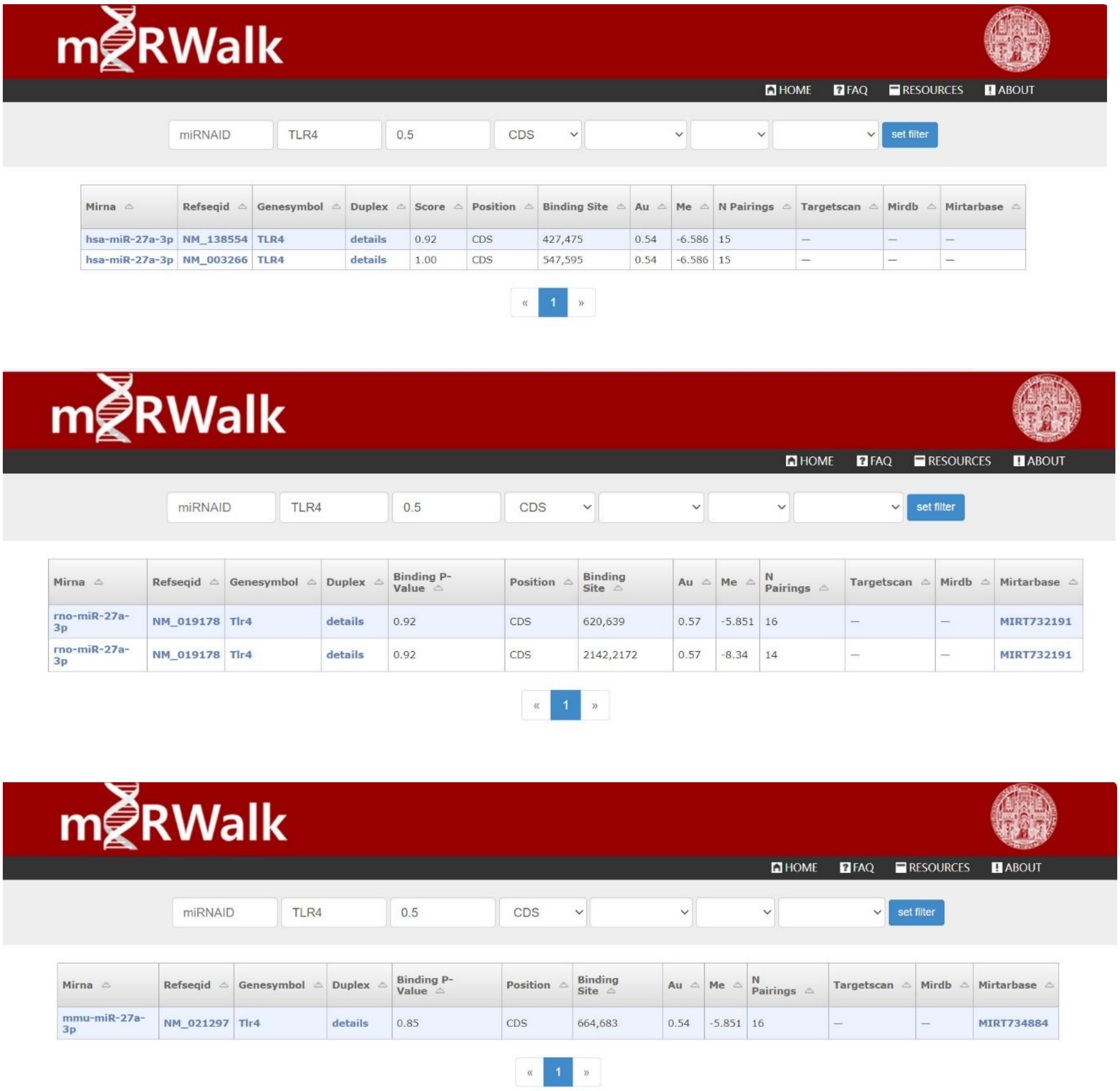

Mirna	Refseqid	Genesymbol	Duplex	Score	Position	Binding Site	Au	Me	N Pairings	Targetscan	Mirdb	Mirtarbase
hsa-miR-27a-3p	NM_138554	TLR4	details	0.92	CDS	427,475	0.54	-6.586	15	—	—	—
hsa-miR-27a-3p	NM_003266	TLR4	details	1.00	CDS	547,595	0.54	-6.586	15	—	—	—

Mirna	Refseqid	Genesymbol	Duplex	Binding P-Value	Position	Binding Site	Au	Me	N Pairings	Targetscan	Mirdb	Mirtarbase
rno-miR-27a-3p	NM_019178	Tlr4	details	0.92	CDS	620,639	0.57	-5.851	16	—	—	MIRT732191
rno-miR-27a-3p	NM_019178	Tlr4	details	0.92	CDS	2142,2172	0.57	-8.34	14	—	—	MIRT732191

Mirna	Refseqid	Genesymbol	Duplex	Binding P-Value	Position	Binding Site	Au	Me	N Pairings	Targetscan	Mirdb	Mirtarbase
mmu-miR-27a-3p	NM_021297	Tlr4	details	0.85	CDS	664,683	0.54	-5.851	16	—	—	MIRT734884

Supplementary Figure 2: Bioinformatics prediction results miR-27a-3p/TLR4 pairs in miRWalk database.

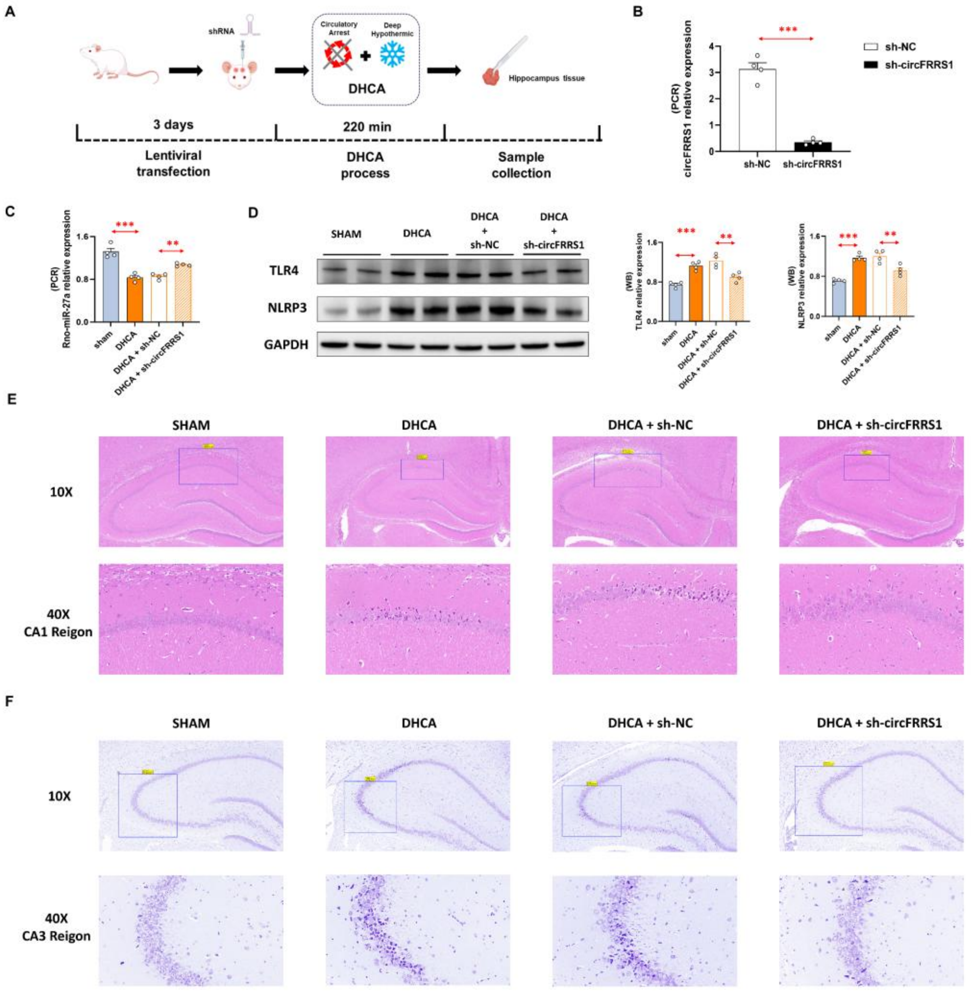

Supplemental Figure 3. Reduced expression of circFRRS1 alleviates hippocampal damage in DHCA rats by regulating the miR-27a-3p/TLR4 pathway.
A. Schematic diagram of the experimental timeline for the in vivo study. **B.** Efficiency of lentivirus-mediated reduced expression of circFRRS1 in the DHCA rat model. **C.** Administration of sh-circFRRS1 could reverse the downregulation of miR-27a-3p expression in DHCA rat hippocampus tissues. **D.** Administration of sh-circFRRS1 could inhibit the overexpression of TLR4 and NLRP3 protein expression in DHCA rat hippocampus tissues. **E.** Representative HE staining revealed lower numbers of necrotic neurons in the hippocampus regions of DHCA + sh-circFRRS1 group than the DHCA + sh-NC group (Enlarged region: CA1). **F.** Representative Nissl staining showed lower shrunken and deeply stained neurons in the hippocampus of DHCA + sh-circFRRS1 group than the DHCA + sh-NC group (Enlarged region: CA3). DHCA: deep hypothermic circulatory arrest. * $p < 0.05$; ** $p < 0.01$, *** $p < 0.001$.
